# Implementation of Covid-19 protection protocols and its implication on learning & teaching in public schools

**DOI:** 10.1016/j.heliyon.2022.e09362

**Published:** 2022-05-02

**Authors:** Anwar Ahmed, Birhanu Sintayehu

**Affiliations:** a School of Education and Behavioral Sciences, Werabe University, Ethiopia; b Department of Educational Planning and Management, Haramaya University, Ethiopia

**Keywords:** COVID-19, Implementation, Learning-teaching, Protection protocols, Public school

## Abstract

This study aimed to investigate the implementation of COVID-19 protection protocols and its implication on teaching –learning process in schools of Werabe administrative town. A concurrent embedded research design and mixed approach were used to carry out the current study and collect data in that order. To conduct the study, 140 teachers, 12 principals, and 6 supervisors were selected by simple random, purposive, and availability sampling techniques respectively. To gather the data, a questionnaire, semi-structured interview, observation checklist, focus group discussion, and document analysis were used. The analysis of quantitative data was carried out by using mean and multiple regressions, while qualitative data was analysed through thematic description and word narration. The results showed that COVID-19 protection protocols were not strictly implemented as per the standards set by both Ministry of Education and the World Health Organization. In addition, the study exposed school community-related factors like reluctance in keeping physical distancing (mean = 4.28, Beta = 0.29, p < 0.05) and resource-related factors like shortage of thermometer (mean = 2.85, Beta = 0.25, p < 0.05) are significantly affecting the implementation of COVID-19 protection protocols. The findings further indicated that the school-community related and the resource-related factors directly affected the classroom teaching-learning process, render students’ families and education sectors for additional financial expenses. Therefore, encouraging communication and coordination among education actors, families, learners, and health communities need to be re-strengthened, and the implementation of protection protocols has to be enforced.

## 1 Introduction

Corona virus is a highly contagious pandemic disease that was initially discovered on 31 December 2019 in China, Wuhan province (World Health Organization [WHO], 2020a) and was officially declared as a global pandemic by the WHO in March 2020 (Karakose et al., 2022). Next to this, similar cases were observed in Thailand, Japan, and the Republic of Korea on 13^th^, 15^th^, and 20^th^ January 2020 respectively (WHO, 2020b).

According to WHO (2020c), the COVID-19 pandemic was reported in Africa for the first time in Egypt on 14^th^ February 2020. Then, on the 25^th^ February 2020, Algeria was the second country to report the first case of COVID-19. In Ethiopia, the first COVID-19 case was reported in Addis Ababa on 13^th^ March 2020 (WHO, 2020d). Subsequently, additional cases were reported in Ethiopian cities and regional states such as Adama, Amhara, and Dire Dawa city administration on 29^th^, 30^th^, and 31^st^ of March 2020 respectively. Three days after the first case was recorded in Ethiopia, the office of the Prime Minister has announced that schools, sporting events, and public gatherings shall be suspended for 15 days. As a result, schools were closed and more than 26 million pre-primary, primary, secondary, and tertiary-level learners from more than 47,000 schools were urged to stay home (Adele, 2020; Mengistie, 2020;; MoE, 2013a).

Sumitra and Roshan (2021) and UN (2020) stated that the COVID-19 pandemic has caused the largest interruption in the education sectors ranging from lower grades to higher education levels which heavily impacted learners, teachers, and parents around the world. The UN report further emphasized that 94% of learners worldwide were affected by the pandemic, representing 1.58 billion children and youth in 200 countries by mid of April 2020. Another negative consequence of the pandemic is that many students have been forced to continue their education online from home following the physical closure of schools and face-to-face lessons (Karakose et al., 2022; Mochida et al., 2021).

After suspension for months, countries in the world started re-opening their schools by setting three major models which were aimed at safe learning and teaching process.

The first model according to Brandon et al. (2020) was affected grade model which is also termed as adjusting physical arrangement by ADEA et al. (2021). In this model, students are stratified based on their grade levels or divided d in the way that classrooms can accommodate smaller sizes and allow the setting of expanded infrastructure like desks and tables. This approach requires extra teachers to handle additional classes. For instance, schools were initially re-opened only for younger children in Belgium, Denmark, France, Greece, Norway, and Sweden while in Germany schools were re-opened only for older students based on the trust that older students would be more able to comply with physical distancing and transmission control measures. Moreover, Cameroon, Kenya, Malawi, Niger, Lesotho, Rwanda, Somalia, and Togo are among the countries that took such measures (ADEA et al., 2021).

The second model of re-opening schools was adjusting class schedules. In this model, alternating shifts and alternating school days were predominantly practiced with the aim of accommodating smaller class sizes and ensuring greater social distancing. For instance, Germany, South Korea, and Scotland have made their students attend alternate shifts (morning and afternoon) while students in Belgium and Switzerland were made to attend alternate days. In addition; Senegal, Gambia, Nigeria, Cameron, Mozambique, Malawi and Somalia are some of the GPE partner countries that used this model (ADEA et al., 2021).

The third model according to Brandon et al. (2020) was implementation of transmission control measures such as class size reduction, physical distancing, face masks, hand washing, temperature checks, and viral or antibody testing. For example Belgium, France, Germany, Israel, Japan, South Korea, Taiwan, and Vietnam applied the use of face masks with some variability in age requirements while Belgium, Denmark, France, Germany, Greece, South Korea, Norway, Scotland, and Switzerland reduced class size (typically 10–15 students or approximately 50% of the normal education period capacity). Some other countries have used desktop dividers to enhance physical separation between classroom desks (Israel, Sweden, Taiwan, and Vietnam). Temperature checks at school entries have been also instituted in some countries (Japan, South Korea, Taiwan, and Vietnam). Summary of school closure and re-opening dates for some selected countries are presented in Table 1 below.

**Table 1:** Summary of school closure and re-opening dates in few selected countries.

No	Continent/region	Country	Date of closure (M/D/Y calendar)	Date of re-opening *(M/D/Y calendar)*
1	Asia	Israel	12 Mar 2020	3 May 2020
		Vietnam	28 Feb 2020	18 May 2020
		Japan	2 Mar 2020	24 Apr 2020
		South Korea	2 Mar 2020	8 June 2020
2	Europe	France	3 Mar 2020	11 May 2020
		Germany	3 Mar 2020	4 May 020
		Belgium	13 Mar 2020	18 May 2020
		Norway	11 Mar 2020	20 Apr 2020
3	America	US	Fall 2020	Varies for states
4	Africa	Ghana	16 Mar 2020	-
		Nigeria	25 Mar 2020	-

**Source**: ADEA et al. (2021)*,*Brandon et al. (2020), and Haidar et al. (2020).

With regard to Ethiopia, the 5th round 3rd emergency meeting was held by the House of People's Representatives of Ethiopia on September 18, 2020. During the meeting, the Minister of Health (MoH) presented a report regarding the COVID-19 pandemic and reported that sexual harassment and early marriage are on the rise in connection with the closure of schools. Strengthening this, ADEA et al. (2021) and WHO (2020e) indicated that school closure in many countries has been associated with unintended teenage pregnancies, sexual exploitation, early forced marriages, violence, and other threats. Considering these and other related violence's, the Minister of the MoH in her speech, recommended that schools need to be reopened in view of the requirements and precautions set by the WHO (Siyanne, 2020). At the meeting, The Minister of MoH Dr. Lia Tadesse said the following;

“To reopen schools which were closed beginning from 13^th^ March 2020, a guideline on COVID-19 protection protocol will be prepared and the task force that foresees the implementation of the guideline will be formed at various levels. She also urged the state to enforce the compulsory wearing of masks, keeping hand hygiene, and physical distancing to be enforced (Siyanne, 2020, Page 3)”.

Following the over mentioned event, survey was conducted by taking 25 districts (the so-called woredas of the Ethiopian administrative system) and 200 schools as a sample from 10 regional states and 2 administrative cities of Ethiopia. The purpose of the survey was to identify the existing gaps in the schools and to prepare a strategic document (guideline regarding COVID-19 protection protocols) that guides the re-opening of schools. Consequently, MoE has prepared a document having organizational structure, standards of schools, roles and responsibilities of various service delivery sectors/offices like education, health, finance, water, women, and youth affairs (MoE, 2013a). After this, successive discussions were made at the federal, regional, zonal, district, and school levels, and agreements were achieved to reopen schools in line with the directions and criteria's’ set by both MoE and WHO (MoE, 2020).

To reopen schools, Ethiopia has used the amalgamation of both the second model (scheduling) and the third model (implementation of transmission control measures). However, priority was given to grade 8 and 12 students who were required to sit for national examinations. This is also in line with the findings of ADEA et al. (2021) which stated that school reopening by the majority of GPE partner countries in Africa like Burkina Faso, Guinea, Kenya, Liberia, Madagascar, Sierra Leone, Zambia and Zimbabwe was done in phased manner in which grades that are expected to sit for national exams are prioritized and re-opened first. Accordingly, schools that are located in both rural and urban areas of Ethiopia that meet the minimum requirement of re-opening were allowed to start their work as of 19 October 2020 and 26 October 2020 respectively (SNNPRS, 2013; Werabe Administrative town, 2013). Likewise, schools in Silte zone including Werabe administrative town where the present study was conducted were re-opened since October 2020.

The present research is worth studying for the following reasons; first, almost no research studies have been directed to examine the implementation of Covid-19 Protection Protocols and their implication on learning & teaching at public schools in the Ethiopian context in general and Werabe administrative town in particular. Second, if Covid-19 Protection Protocols are implemented improperly and problems remain unsolved, schools would fail to meet their mission and vision (Sintayehu and Hussien, 2021). Third, though school communities are increasingly vaccinating, however Covid-19 pandemic still exists. On the other hand, closed schools are reopening everywhere. In these circumstances, proper the implementation of Covid-19 protection protocols during learning & teaching in schools is of paramount importance since school stakeholders play a key role in the continuation of teaching-learning throughout the epidemic, have undertaken essential responsibilities (Karakose et al., 2022). Fourth, the findings of the study may pinpoint the major gaps and show direction to rectify to existed glitches in implementation of Covid-19 Protection Protocols.

## 2 Review of related literature

Currently, numerous researches have been conducted on the area of COVID-19. However, the majority of them have focused on the impact of the pandemic. For instance, Sumitra and Roshan (2021) and Suzanne (2021) identified that COVID-19 pandemic has resulted in loss of instructional time delivery and negatively affected the academic performance of students. Additionally, ADEA et al. (2021), save the children (2020), UN (2020), and Yorke et al. (2020) found out that COVID-19 pandemic has enhanced the rate of students’ dropout. Moreover, the COVID-19 pandemic has affected the internal assessment and examination strategies (Sumitra and Roshan, 2021); increased the risk of gender-based sexual harassment, violence and abuse (Save the children, 2020; Suzanne, 2021; UNESCO, 2020; UNICEF 2020); created challenge in mental health and wellbeing of staff and students (Suzanne, 2021); increased the cost of the necessary resources like gloves and masks used to prevent the spread of COVID-19 (Yorke et al., 2020); created fear among children and their teachers not to go back to school due to the safety issues (ADEA et al., 2021); increased risk on schooling access and quality especially for girls and other vulnerable groups (ADEA et al., 2021; UNESCO, 2020). According to Haider et al. (2020), COVID-19 pandemic has impacted countries by reducing the number of tourists which in turn negatively affected their economy, reduced agricultural productivity which further exposed humans to food insecurity, and brought negative political consequences.

Despite the negative consequences discussed so far, COVID-19 pandemic has brought some positive aspects that benefited human life. For example, COVID-19 pandemic has created an opportunity for the reduction of greenhouse gas emissions and other pollutants, lowered the incidence of traffic accidents, helped some families to spend more time together (Haider et al., 2020). In the same way, National Governors Association [NGA] (2020) and Sumitra and Roshan (2021) found out that the lockdown due to COVID-19 pandemic has encouraged hybrid learning opportunities (mix of distance and in-person learning). Moreover, NGA (2020) explained that virtual learning, e-learning, and online learning opportunities were part of distance learning that gained attention due to the occurrence of the COVID-19 pandemic. Andreas (2020) on his part expressed that the COVID-19 pandemic has created an additional opportunity for teachers to receive professional training in the utilization of ICT to cope up with the virtual and online teaching mechanisms.

On the other hand, studies conducted by Andreas (2020), Giuseppina et al. (2020), Minnesota department of health (2021), MoE (2013a), MoSHE, 2013), Shelbay et al. (2020) and Rachael et al. (2020), and Werabe administrative town (2012) have focused on the mitigation strategies rather than the negative and positive impact of the covid-19. According to them, the mitigation strategies while reopening schools include temperature checks, hand sanitizing, wearing masks, social/physical distancing between students and staff, limiting classes size to small cohorts of students, regular cleaning, promoting remote learning, outdoor learning, building more classrooms and upgrading school buildings. Moreover, Claire and Lindsay (2020) advised that schools in Taiwan and Hongkong are required to use plastic barriers in classrooms in order to increase students’ separation in addition to wearing masks, checking body temperature using a mobile app, or a contactless thermometer at the school entrances.

Generally, the above mentioned studies have highlighted insight about the impact of COVID-19 and its mitigation strategies which shows that these studies were inadequate to address the concerns in relation to the implementation of COVID-19 protection protocols against the set standards by both MoE and WHO. Therefore, this research intends to investigate the implementation of COVID-19 protection protocols and its implication on learning & teaching at public schools of Werabe administrative town.

### 2.1 Objectives of the study

The specific objectives of this study were to:

a) Investigate the extent to which schools implement COVID-19 protection protocols with reference to the standard set by MoE and WHO;
b) Determine the major challenges that affect the implementation of COVID-19 protection protocols
c) Identify which factor (school community-related or resource-related factor) is the most statistically significant in affecting the implementation of COVID-19 protection protocols

## 3 Research methodology

### 3.1 Research design

A mixed method design that involves both qualitative and quantitative methods was employed to carry out this study. Specifically, the concurrent embedded type that allows data integration (combination of both quantitative and qualitative data) at the time of data collection by giving priority to one of the elements and nesting the second in the first element was used (Creswell, 2009). To collect the required data, the researchers consulted both primary and secondary sources of data. The primary sources of data were teachers, students’, unit leaders, principals, supervisors, committee members of the COVID-19 protection task force, and education sector heads. As secondary sources, various documents particularly MoE guidelines, journal articles, WHO reports, attendance sheets, lesson plans, and various reports developed by Werabe administrative town were considered.

### 3.2 Context of the study

Based on the decision made by the Federal Government of Ethiopia, schools in urban areas and rural areas that, fulfilled the minimum standards of re-opening which were set by MoE, have been re-opened since October 19 and 26, 2020 respectively.

At the same time, schools in Silte zone particularly Werabe administrative town where the present study was conducted were re-opened. Following this, the researchers collected the data from 13 December 2020 to 20 February 2020. Both qualitative and quantitative data were collected side by side by strictly applying the appropriate COVID-19 protection protocols like social distancing, waring face masks and other protection strategies in order to reduce the risk of COVID-19 transmission from both parties (researchers and data providers).

### 3.3 Study group/participants

According to Silte zone annual abstract (2012), Werabe administrative town has 27 primary schools and 3 secondary schools. The total population of this study was 15,738, of which 15,201 are students, 490 are teachers, 41 are principals, and the remaining 6 are supervisors. Among the 30 schools available in the town, 15 (50%) of the schools are selected by using a simple random sampling technique. In the selected schools, there were 9576 students, 309 teachers, 22 principals, and 6 supervisors. Hence, 12 students, 140 teachers (45%), 12 principals (54%), and 6 supervisors (100%) were selected by using simple random, purposive, and availability sampling techniques respectively. In all these stages, the sample selection is based on the sampling principle advised by Mugenda and Mugenda (2003) which states that it is adequate to take 10%–30% or more of the respondents that represent the target population. Regarding the sampling techniques, simple random sampling was used to select teachers with the aim of giving equal chance of inclusion while purposive sampling was used to deliberately include the main principals as they are responsible to lead COVID-19 related activities rather than the vice principals (Sintayehu, 2020).

### 3.4 Data gathering tools

Regarding data collection tools; questionnaires, interviews, document analysis, focus group discussion, and observation checklist were used. A questionnaire, both close and open-ended type, was designed to congregate data from teachers. It had two sections. The first section dealt with the background information of the respondents. The second section was related to the implementation of COVID-19 protection protocols and the challenges that affect the implementation of the protection protocols. The questionnaire comprised of 6 items on the background information of respondents, 15 items about the implementation of COVID-19 protection protocols, and 20 items about challenges that affect the implementation of the protection protocols. All the items were adopted by the researchers in line with available literature and COVID-19 protection protocols developed by MoE. The items regarding the implementation of COVID-19 protection protocols had 5 point scales, ranging from 1- almost never to 5 - always. The second part of the items, which were concerned with the challenges that affected the implementation of COVID-19 protection protocols, was Likert types rated on a scale of 0–4 (0- not at all: 1-to some extent: 2-moderately: 3-highly: and 4- extremely).

To ensure the validity and reliability of the questionnaire, a pilot test was conducted on 38 randomly selected teachers who were not members of the sampled schools. Accordingly, the internal consistency reliability of 0.87 is obtained for items related to the implementation of COVID-19 protection protocols and 0.82 for items related to the challenges that affect the implementation of the protection protocols which are acceptable according to Cohen et al. (2007).

A semi-structured interview was conducted with principals, supervisors, unit leaders, and education sector heads to gather data regarding the formation, functionality, and coordination of the COVID 19 protection task force. Additionally, data regarding the implementation of COVID-19 protection protocols and major challenges regarding its implementation were collected by using interviews. An audio recorder was used to capture the interview data. The interview was conducted in English at second cycle primary and secondary schools taking into consideration that the respondents are capable enough to express their ideas as English is the medium of instruction in their school while Amharic was used at first cycle primary schools.

Moreover, two sets of FGD having 8–12 members were conducted in each school. The first set was conducted with committee members of the COVID-19 protection task force while the second set was done with students. The rationale behind FGD was to gather data regarding the roles of teachers, students, task force on the implementation of the COVID-19 protection protocol. Moreover, it targeted the major challenges that affect the implementation of the COVID -19 protection protocols.

Documents like MoE guidelines, reports developed by WHO and Werabe administrative town, journal articles, attendance sheets taken from homeroom teachers, and lesson plans were used to collect the data in a way that supplements the data collected through questionnaires. The attendance sheet taken from homeroom teachers was analyzed to see whether the numbers of students assigned per classroom are in line with the criteria set by the protection protocol and WHO. Moreover, weekly lesson plans prepared by teachers were checked to see whether or not teachers are using appropriate teaching and assessment methods that do not expose students to the transmission of COVID-19.

By using an observation checklist developed by the researchers, direct observations of the classroom situation and school environment were conducted to strengthen the data collected through the above-mentioned instruments. Regarding classroom situations; sitting arrangement, students’ carefulness in wearing face masks, availability of sufficient chairs, desks, etc were focused. Regarding the school compound, the observation was taken place focusing on elements components like separation of entry and exit gates, availability of water, sanitizers, library usage, mask-wearing, and other issues.

### 3.5 Data analysis

Descriptive statistics (mean, median, and mode) were used to analyse quantitative data collected through the questionnaire. Inferential statistics (multiple regressions)were also applied to witness which of the identified factors affecting the implementation of COVID-19 protection protocol are statistically significant. Moreover, qualitative data that was secured through an interview, FGD, and document analysis were analyzed thematically through narration. Prior to conducting regression analysis, the assumptions of independence, normality, and homogeneity were checked. Kolmogorov-Simrnov and Shapiro-Wilks’ tests were conducted to check the normality of data collected from different institutions/schools. Both tests as indicated in Table 2 below showed that sample data were generated from normally distributed population as their p value is greater than 0.05.

**Table 2:** Tests of Normality.

	Kolmogorov-Smirnov^a^			Shapiro-Wilk		
	Statistic	Df	Sig.	Statistic	Df	Sig.
Schools	.177	140	.200∗	.875	140	.064

a Lilliefors Significance Correction

∗ This is lower bound of the true significance.

### 3.6 Research ethics

Ethical approval was obtained from Werabe University research and ethical board. In approaching the individual participants, negotiation was made first by informing the purpose of the study and taking into account their interest or willingness to participate. Moreover, the respondents were assured about the confidentiality of their response that it will be used only for research purpose and not passed to the third party. Furthermore, the researchers firmly applied all the proper COVID-19 protection protocols such as social distancing, the use of face mask and disinfecting chemicals like sanitizers while approaching participants at the time of data collection.

## 4 Results and discussions

In line with the research objectives, the major findings and discussions are presented here under.

### 4.1 Research objective #1: implementation of COVID-19 protection protocols with reference to the standard set by MoE and WHO

*Implementation of COVID 19 protection protocol:* According to MoE (2013a), and Werabe administrative town education office (2013) schools should fulfil the set standards before re-opening. For instance, it is mandatory for all students and teachers to wear face masks, schools need to allocate one desk per a student, the school should accommodate a maximum of 1000 students in one shift, staff and students need to keep 2-meter distance in the school environment, schools have to provide sufficient sanitizer, cleaning water and soap as per the size of their students. Table 3 shows the response of respondents regarding the implementation of COVID-19 protection protocol.

**Table 3:** Implementation of COVID-19 Protection Protocol.

No	Items To what extent do schools	Descriptive Statistics					
		N	Min	Max	Mean	median	Mode
1	Provide awareness about the impact of COVID-19 for the students and staff through discussion forums	141	.00	4.00	2.74	3.0	3.0
2	Applied the appropriate physical distancing principles (minimum 1 m)	140	.00	4.00	2.39	2.0	2.0
3	Ensured appropriate use of masks where physical distancing cannot be maintained	139	.00	4.00	2.38	2.0	2.0
4	Clean and disinfect frequently touched surfaces such as door handles, desks, toys, supplies, light switches, doorframes, play equipment, teaching aids used by children and covers of shared books.	140	.00	4.00	1.97	2.0	2.0
5	Allocate only 20–25 students per classroom	139	.00	4.00	3.35	4.0	4.0
6	Applied the principle of “one student per desk”	136	.00	4.00	3.44	4.0	4.0
7	Availed sufficient water to help students and staff to keep themselves clean (hygiene)	141	.00	4.00	2.52	3.0	3.0
8	Prepared thermometer to measure the body temperature of staff at the gate or entrances	138	.00	4.00	.91	0.0	0.0
9	Practiced one book per student principle	134	.00	4.00	1.72	2.0	0.0
10	Differentiated the gates (entrances) to allow students to get in and out (If shifts are available) or differentiated exit and entrance gate	139	.00	4.00	2.29	2.0	3.0
11	Made classrooms to have windows that ventilate sufficient air to avoid suffocation	143	.00	4.00	3.01	3.0	4.0
12	Prepared alternative (extra) classrooms to overcome the challenges if symptoms of COVID-19 is happened	141	.00	4.00	2.18	2.0	4.0
13	Established clear information and feedback sharing mechanisms with parents, students and teachers	141	.00	4.00	2.37	3.0	3.0
14	Provide sufficient soap and clean water or alcohol-based rub at school entrances and throughout the school	142	.00	4.00	2.31	2.0	2.0
15	Designed continuous follow up or support mechanisms concerning to COVID 19	142	.00	4.00	2.19	2.0	2.0
**Overall mean**					**2.38**		

Scales of interpretation <0.5-almost never; 0.5–1.49-rarely; 1.5–2.49-sometimes; 2.5–3.49-usually; 3.5-4-almost always.

As depicted in Table 3 above, the overall computed mean score of all respondents was 2.38. This indicates that COVID-19 protection protocol is not strictly implemented as per the standard set by MoE and WHO. Similarly, students at the time of FGD elaborated that the overall implementation of the protection protocol is low particularly in availing thermometer and differentiating entry and exit gates. Specifically, the mean score of item numbers 4, 8, and 9 is even below the average mean score (2), which indicates that the implementation of the activities indicated on these items is by far less than the standard developed by MoE. For instance, the mean score of 1.97 (item 4) tells that frequently touched surfaces like door handles, desks, toys, supplies, light switches, doorframes, play equipment, teaching aids used by children, and covers of shared books are rarely cleaned & disinfected in the study area. In connection with this, Lisa (2020) stated that schools should enforce regular hand washing with safe water and soap, alcohol rub/hand sanitizer or chlorine solution at a minimum, daily disinfection, and cleaning of school surfaces. About cleaning surfaces, one of the interviewed school principals (SP) complained as follows;

As you know, our school is a public school in which children from relatively poor families and those who have less awareness about the benefits of education are attending. Due to this, the government has provided 2 masks for each student at the beginning of reopening. Our school has a financial problem to buy sanitizers and disinfectants. As a result; door handles, desks and play equipment are not frequently disinfected [SP2; on 12 February 2020].

Regarding body temperature measuring devices, documents like (MoE, 2013a; MoSHE, 2013) encourage schools to have a thermometer at the gate so that the body temperature of all staff, particularly the students can be checked when they go in and out of the school compound. However, the mean score of 0.91 (item 9) indicates that schools do not have the device and students pass the school gate without measuring their body temperature. Confirming this, the observation checklist used for data collection indicated that all government schools (12 observed) do not have a thermometer. Furthermore, the mean score of 1.72 for item number 9 indicates that the implementation of one book per student principle is not sufficiently realized in the study area. In connection with this, one of the interviewed SP said;

In our school, most books are given to an average of three students. Although the protection protocol recommends one book for one student, it is difficult to address. Some books like Siltie language in grade 6 are given for a group of 6 students [SP4; 27 February 2020].

The mean score of item numbers 2, 3, 10, 12, 13, 14, and 15 is between 1.5 and 2.49. This implies that activities like appropriate physical distancing (item 2), appropriate use of masks in places where physical distancing cannot be maintained (item 3), differentiating entry and exit gates (item 10), and building extra classrooms (item 12) are not strictly implemented as COVID-19 protection protocol. Nevertheless, Andreas (2020) stated that social distancing of 1–2 m between pupils and staff has proven to be one of the most effective measures to prevent the spread of the COVID-19. He further explained, schools in less-affected areas in Japan are required to maintain a distance of 1 m while those in more affected ones must maintain a distance of 1–2 m. In addition, MoE (2013a), MoSHE (2013), and Werabe administrative town (2012) stated that 1-meter physical distancing principles should be strictly implemented to protect COVID-19. Similarly, a study conducted by the Minnesota Department of Health (2021) stipulated that those teaching a class or course must wear a face covering or face shield unless a physical distance of 6 feet or greater can be maintained at all times by students in their class. Regarding face masks (item 3), Andreas (2020), MoE (2013a), MoSHE (2013), Werabe administrative town (2012), the Minnesota Department of Health (2021) stated that staff and faculty must wear face coverings when working outdoors in areas where a social distancing of 6 feet or more cannot be maintained. Regarding the provision of sufficient soap and clean water or alcohol-based rub at school (item 14), Andreas (2020) found out that corona virus mitigation strategies such as good hygiene, and regular cleaning are a must to reduce its risk.

The mean scores of activities indicated in item numbers 1, 5, 6, 7, and 11 are between 2.5 and 3.49. This shows that the activities indicated in these items are usually implemented in the study area. Specifically, the mean score of 1.74 (item 1) tell us that schools usually provide awareness about the impact of COVID-19. Supporting this, almost all school principals and teachers had received information about COVID-19 and this was received mainly through the television, face-to-face communication, by radio and by phone. He also added the types of information they received included practical information about hand washing, physical distancing and staying at home and wearing a facemask (Sintayehu and Adem, 2020; Yorke et al., 2020).

Regarding the allocation of students per classroom, item number 5 (mean = 3.35) tells that schools have usually allocated 20–25 students per classroom, which is in line with the MoSHE (2013) guideline. However, some countries like France and the United Kingdom have recommended a limit of 15 students in primary classes in order to provide the safety distances are maintained (Andreas, 2020). Astonishingly, data obtained through direct classroom observation indicated that schools have an average of 30–35 students per class, which is against the directions stated in the protection protocol, and WHO. This number is also against of Andreas (2020) limit which dictates that countries like Chile, Colombia and Japan that have more than 30 students per class in lower secondary level will face more difficulties in reorganising classes into smaller groups of students to maintain a safe distance between desks. Supporting the present finding and opposing the findings obtained through a questionnaire, one of the school supervisors (SS) at the time of interview reported that “Due to the large number of students we had before the occurrence of COVID-19, we have been forced to violet the principle of COVID-19 protection protocol and allowed 30–35 students to attend lesson in one section” [SS1; 13 January 2020]. Similarly, data collected through document review, particularly Werabe administrative town (2012) evidenced that all schools available in the study area except Mariyam school require the construction of additional classes (a total of 342) to implement 30 students per class principle. This indicates that the principle of implementing 20–25 students per class in the study area was not realized as per the standard set by MoE. Moreover, attendance sheets taken from homeroom teachers indicated that almost all schools have more than 30 students per classroom.

Regarding the principle of implementing one student per desk, the mean score of item number 6 in Table 3 is 3.44 which tell us that this principle is usually applied in schools. Unexpectedly, data collected through observation showed an opposite result. Meaning, more than 90% of the schools do not apply the one student per desk principle. The researcher observed that an average of two students is sharing common sits in the majority of the observed schools. In addition to this, a document reviewed (Werabe Administrative town, 2012) indicated that schools require extra/additional desks (a total of 3773) to implement one desk per student principle. In this regard, the unit leader (UL) of school X at the time of interview expressed as follows;

I am comfortable with the sitting arrangement made after the incidence of COVID-19. Before that three students were used to sit at a single table which was suffocating and created an opportunity for students to disturb one another. Now, maximum of two students are sharing a common table in order to reduce the transmission of COVID-19[UL2; 13 December 2019].

Similarly, another interview held with the school supervisor confirmed that two students are made to sit in one chair due to the occurrence of COVID-19 and this by itself has brought good opportunities in reducing misbehaviour. The supervisor further explained that the present sitting arrangement is playing a great role in reducing cheating opportunities at the time of the exam.

Furthermore, the mean score of item number 11 is 3.01, which is between 2.5 and 3.9, which indicates that the practice of ventilating classrooms to have sufficient air and avoid suffocation is usually practiced in the study area. Moreover, data collected through observation repeated the same. Meaning, almost all schools have open windows that allow sufficient air to blow into the classrooms. Similarly, Giuseppina et al. (2020) stated that ventilation should be ensured before entrance, during break, at the beginning and ending of classes/day and every 3 h. He added that ventilation should last at least 15 min and windows and/or doors should be kept open.

In Table 3 above, item number 11 has negatively skewed result as its mean (2.98) is less than the mode (4.00) and the median (3.00) that lies in between them.

To sum up, the findings in Table 3 above indicates that schools in the study area have utilized the three models of school re-opening discussed under introduction part. For instance, differentiating entry and exit gates (item 10) refers the second model of school re-opening (alternation of school day/shift) while reducing the number of students in class (item 5), allocating one student per desk (item 6), and building extra classrooms (item 12) refers the first model of school re-opening which was referred as adjusting physical arrangement. Moreover, schools of the study area have used the third model of school reopening named as using transmission control methods like physical distancing (item 2), wearing mask (item 3), and using thermometer (item 8).

### 4.2 Research objective #2: major challenges that affect the implementation of COVID-19 protection protocols

For the sake of convenience, factors affecting the practice of COVID-19 protection protocols are classified as school community-related and resource-related factors. Tables 4 and 5 show their summary respectively.

**Table 4:** Resource related factors.

Item no	Description: To what extent do the following factors challenge the practice of COVID-19 protection protocols?	Descriptive Statistics					
		N	Min	Max	Mean	Median	Mode
1.	Scarcity of chemicals to clean the school	142	1.00	5.00	2.59	3.00	1.00
2.	Shortage of mask to cover the mouth and nose	143	1.00	5.00	2.76	3.00	3.00
3.	Shortage of materials like soap, alcohol sanitizer	140	1.00	5.00	2.87	3.00	3.00
4.	Shortage of water	141	1.00	5.00	2.59	2.00	1.00
5.	Deficiency of body temperature measuring device (Thermometer at gates)	141	1.00	5.00	2.85	3.00	1.00
6.	Shortage of textbooks to apply the principle of one book for one student	141	1.00	5.00	2.75	2.00	2.00
7.	Shortage of classrooms	141	1.00	5.00	2.49	2.00	1.00
8.	Shortage of chairs and desks	142	1.00	5.00	2.70	2.50	1.00
9.	Shortage of books to practice one book one student principle	142	1.00	5.00	2.71	3.00	2.00
10.	Uncomfortable classroom structure to ventilate air	143	1.00	5.00	2.71	3.00	1.00
11.	Shortage of teachers	143	1.00	5.00	2.32	2.50	1.00
***Overall mean***					***2.67***		

Scales of interpretation <0.5-very slightly; 0.5–1.49-Slightly; 1.5–2.49-Moderetly; 2.5–3.49-Highly; 3.5-4-Very highly.

**Table 5:** The output of multiple regressions analysis.

Model Summary				
Model	R	R Square	Adjusted R Square	Std. Error of the Estimate
1	.256^a^	.066	.056	10.49898

ANOVA^b^						
Model		Sum of Squares	Df	Mean Square	F	Sig.
1	Regression	1464.773	10	146.477	1.292	.248^a^
	Residual	9978.641	88	113.394		
	Total	11443.414	98			

Coefficients^c^						
Model		Unstandardized Coefficients		Standardized Coefficients	T	Sig.
		B	Std. Error	Beta		
1	(Constant)	26.260	3.280		8.006	.000
	Item 1	1.763	1.131	.218	1.560	.122
	Item 2	.125	1.235	.015	.102	.919
	Item 3	-.330	1.088	-.043	-.304	.762
	Item 4	.452	.930	.058	.486	.628
	Item 5	1.567	.695	.252	2.255	.027
	Item 6	-1.534	1.241	-.210	-1.236	.220
	Item 7	-.001	1.415	.000	.000	.999
	Item 8	.691	1.292	.093	.535	.594
	Item 9	1.395	1.494	.176	.934	.353
	Item 10	-.872	1.075	-.114	-.811	.420

a Predictors: (Constant), resource related factors

b Dependent Variable: Implementation of COVID-19 Protection protocols

c Dependent Variable: Depend

As indicated in Table 4 above, the overall mean score of all items in general and the mean score of individual items, in particular, are between 2.5 and 3.49, which indicate that the above-mentioned factors are highly affecting the implementation of the COVID-19 protection protocol. Specifically; shortage of materials like soap, alcohol, sanitizer (item 3, mean = 2.87) indicates that the shortage of these resources is highly affecting the implementation of protection protocols. Supporting this, UNESCO (2021) depicted that guaranteeing schools clean and disinfected; and guaranteeing access to washbasins should be given priority to safely return students and staff to schools. Similarly, Giuseppina et al. (2020) found out that cleaning and disinfecting floors, surfaces, and all premises with alcohol or sanitizer should be done at least once per day with additional cleaning on frequently touched surfaces like door handles. He also stated that toilets must be cleaned frequently at least two or three times a day.

Regarding scarcity of resources particularly body temperature measuring device, Table 4 (item 5, mean = 2.85) shows that its shortage is highly affecting the implementation of the protection protocol. Confirming this; an interview held with one of the school supervisors seems;

COVID-19 protection protocol insists schools to have a thermometer so that the body temperature of students can be checked at the gates. Because of this, we have requested the town administration several times. However, they are unable to buy due to the shortage of budget [SS6, 20 December 2020].

Similarly, students at the time of FGD critically rose that entering schools without checking their body temperature due to absence of thermometer in their school has created a great fear. Supporting the above findings, ADEA et al. (2021) described that GPE partner countries in Africa has experienced inadequate financing, gaps in infrastructure for spacing and hygiene while reopening schools.

Concerning the utilization of mask to cover mouth and nose, item 2 (mean = 2.75) shows that scarcity of mask in schools of the study area is highly affecting the implementation of COVID-19 protection protocols. In the same way, National Academy of Science and Engineering (2020) discussed that dearth of Personal Protective Equipment (PPE), such as masks and face shields for students and teachers; shall be a barrier in school reopening. Supporting this, principal of school Y said;

At the beginning of the school year, the regional state of SNNPRS has provided two masks for each student. These masks have been used for the past 6 months and many of the students are still using them. The problem is that some students have lost them due to several reasons and some are wearing tired masks. As a result, many students are not using the mask in schools [SP1, 24 December 2020].

Similarly, education bureau head at the time of interview confirmed as follows;

We have tried a lot to fulfil requests in relation to masks, water, alcohol, chairs, and others. However, it is very difficult to cover all these expenses as our sector has the shortage of budget. Due to this we have allowed schools to buy the required resources by using their internal incomes [H3, 24 December 2020].

In supporting the above findings, ADEA et al. (2021) described that GPE partner countries in Africa has experienced inadequate financing, gaps in infrastructure for spacing and hygiene while reopening schools.

Item 10 (mean = 2.7) in Table 4 shows that uncomfortable classroom structure to ventilate fresh air is highly affecting the implementation of protection protocol. Correspondingly, National Academy of Science and Engineering (2020) stated that poor-quality school buildings those that have bad indoor air quality and inadequate bathroom facilities can complicate school reopening in America and may make it difficult for school districts to implement the recommended health and safety measures which in turn poses a problem for equitable implementation of the strategies as children and youth from low-income families are disproportionately attending their schools with poor quality facilities. In the same way UN (2020) elaborated that schools must be cleaned and disinfected to inform hygienic practices for students and staff, especially in case they were used as health centres during the closure period.

Table 4 above also shows that shortage of water (mean = 2.59), shortage of classrooms (mean = 2.49), shortage of teachers (mean = 2.32), and shortage of chairs & desks in classrooms (mean = 2.71) are affecting the implementation of COVID-19 protection protocols. Supporting this, UNESCO (2020) stated that around 1.3 million students at the primary level in Latin American Countries (LAC) do not have access to drinking water in their households nor in school. It is urgent that LAC countries invest in the improvement of the state of school infrastructure to offer basic sanitation and hygiene conditions. In addition to this, ADEA et al. (2021) witnessing documents in Ethiopia and Lesotho found out that inadequate access to running water and water points in schools has impeded compliance with requirements for frequent hand washing. Moreover, a report by UNICEF and the (WHO, 2021) estimated that only 44% of children in Sub Sahara Africa had access to water in schools in 2019. In Guinea-Bissau and Niger, only 12% and 15% of schools respectively had basic hand washing facilities (Bisin and Thompson, 2020).

Moreover, the reviewed documents, particularly Werabe Administrative town (2012) indicated that, 3773 additional desks are required to practice one student per desk principle. In addition to this, the same document showed that all schools found in the administrative town except Maremiya school require additional classes and blackboards to apply 30 students per class principle. Specifically, the document showed that 101 additional classes with 101 blackboards are needed to implement the 30 students per classroom principle. Supporting this idea, Carbine's (top management) decision passed on the letter dated 28/01/2013 E.C showed that 850 chairs, 600 masks, and 40 blackboards, 100 sanitizers, and employment of 18 new teachers were needed for the proper implementation of COVID-19 protection protocol (Sintayehu and Menber, 2019). Supporting this, ADEA et al. (2021) wrote that pre-existing challenges with infrastructure especially in schools that are already overcrowded is widespread challenge in GPE member African countries. ADEA further found out that Ghana, Nigeria and Rwanda have average class sizes of 39, 51 and 43, respectively. To meet the need for social distancing, Ghana is moving learning outdoors while Nigeria is splitting classes and Rwanda is adding more classrooms (Ananga and Tamanja, 2017; Rwanda, Ministry of Education, 2019; Statistica, 2018).

Table 4 above further indicates that, item number 4 (mean = 2.6, median = 2.0, Mode = 1.0), item number 7 (mean = 2.6, median = 2.0, Mode = 1.0), and item number 8 (mean = 2.7, median = 2.5, Mode = 1.0) have positively skewed results as their mean value is greater than the mode having the median in between them.

Overall, shortage of resources which are discussed in Table 4 above and required to implement the protection protocol of COVID-19 have negatively impacted financial problem in education sector across all levels (woreda, zonal, regional and federal). Meaning, the sector has lost extra money in fulfilling required materials like soap, sanitizer, face mask, desks, chairs, ventilating classrooms and building additional classrooms. In addition to this, COVID-19 has positively impacted the school environment by helping students and staff to keep their hygiene through the regular hand washes.

### 4.3 Research objective #3: determine most statistically significant factor that affecting the implementation of COVID-19 protection protocols

Multiple regression analysis was conducted to determine which of the resource-related factors is significantly affecting the implementation of the COVID-19 protection protocol. As indicated in Table 5 below, the model summary Table indicates that only 6.6% of the overall implementation of COVID-19 protection protocol is affected by resource related factors. The value of the ANOVA Table (P > 0.05) shows that the combined effect of the 10 resource-related items on the overall implementation of the COVID-19 protection protocol was not statistically significant. More specifically; the coefficients Table indicates that item number 5 (deficiency of body temperature measuring device) is statistically affecting the implementation of COVID-19 protection protocol as its p value is p < 0.05.

As indicated in Table 6 above, the overall mean score of all items (2.97) is between 2.5 and 3.49. This implies that the above-mentioned factors are highly affecting the implementation of COVID-19 protection protocols. Particularly; school community reluctance (mean = 3.95), inability to keep physical distancing (mean = 4.43), carelessness in using masks (mean = 4.23), and type of play practiced by students (mean = 3.87) are the first four ranking factors that affect the implementation of COVID-19 protection protocols. Supporting school community reluctance, Anwar et al. (2016) found out that teacher's commitment to their students, the community and their profession were low due to low salary and low respect by the community. Birhanu and Anwar (2021) also stated that rising of living cost, inadequate monthly salary and derisory housing allowances has resulted low morale of teaching staff. Similarly, an interview held with one of the school principals evidenced the following;

In our school, the carelessness and reluctance of the students and even teachers are seriously affecting the implementation of COVID-19 protection protocols. It is very difficult to control students, particularly during break time. They simply rush outside and form a group by forgetting the 2m distance-keeping principle. Surprisingly, teachers themselves do not appropriately wear masks [SP3, 20 December 2020].

**Table 6:** School community-related factors affecting the implementation of COVID-19 protection protocols.

*No*	*To what extent do the following factors challenge the implementation of COVID 19 protection protocols?*	Descriptive Statistics					
		N	Min	Max	Mean	*Median*	*Mode*
1	Lack of awareness about COVID 19	*143*	*1.00*	*5.00*	*1.48*	*3.00*	*3.00*
2	School community reluctance towards COVID 19 protocols	140	1.00	5.00	3.95	*3.00*	*3.00*
3	Inability to keep physical distancing	141	1.00	5.00	4.43	*2.00*	*2.00*
4	Carelessness in using masks	142	1.00	5.00	4.23	*3.00*	*1.00*
5	Teaching methods used by teachers in classrooms	139	1.00	5.00	2.48	*3.00*	*3.00*
6	Assessment techniques practiced by teachers	140	1.00	5.00	2.41	*3.00*	*3.00*
7	Types of play practiced by students	140	1.00	5.00	.87	*2.00*	*2.00*
8	Absence of awareness-raising mechanisms by the school management	141	1.00	5.00	1.49	*2.00*	*2.00*
9	Absence of progress follow-up concerning the practice of protection protocols	142	1.00	5.00	2.36	*3.00*	*3.00*
***Overall mean***					***2.97***		

Scales of interpretation <0.5-very slightly; 0.5–1.49-Slightly; 1.5–2.49- Moderately; 2.5–3.49-Highly; 3.5-4-Very highly.

Supporting the above findings, data collected through document analysis, particularly lesson plan review, indicated that some teachers are still using group discussion methods for their classroom instruction in a way that exposes students to COVID-19 transmission and violet with the principle of physical distancing. This is also in line with Anwar et al. (2018) which states that group discussion is usually used method of teaching in classroom. Moreover, FGD held with students showed that the majority of students are reluctant to regularly wear masks and do not keep the principle of physical distancing while playing outside the classrooms. Furthermore, the observation checklist conducted in 12 schools indicated that the majority (more than 60%) of the students in the observed schools does not wear masks and unable to keep physical distancing principles both inside and outside the classrooms through the classroom practice is better than outside. In opposite to these findings, government guidelines like (Werabe Administrative towns, 2013; MoE, 2013a; MoSHE, 2013) enforce all teachers and students to regularly wear masks and keep appropriate physical distancing.

Table 6 above also indicates that the teaching method practiced by teachers (mean = 2.48), the assessment technique used by teachers (mean = 2.41), and the absence of progress follow-up (mean = 2.37) are moderately affecting the implementation of COVID-19. However, the absence of awareness about COVID-19 (mean = 1.4944) is less likely to affect the practice of the COVID-19 protection protocol. In the slightly opposite way, a study conducted by Desalegn et al. (2020) found out that one-third of the study participants had poor knowledge regarding COVID-19. They also specified that male gender, age above thirty-five years, lack of formal education, being a farmer, daily laborer, merchant, and housewife was significantly associated with poor knowledge.

The mean score of item number 1 in Table 6 above is 1.48 which indicates that lack of awareness among school community regarding COVID-19 is slightly affecting the implementation of the protection protocol. This indicates that majority of the students have awareness about COVID-19. In line with this, Thinley et al. (2021) found out that 88 (74%) of medical student in Bhutan have good knowledge, 28 (23%) had satisfactory knowledge and only four (3%) had poor knowledge regarding COVID-19. Moreover, students at the time of FGD, rose that they are in a good level of awareness regarding, the symptoms and COVID-19 protection strategies. Furthermore, ADEA et al. (2021) found out that parents’ and teachers understandable fear of infection are some of the overcoming barriers that hinder school reopening.

Multiple regression analysis was conducted to determine the effect of school community-related factors on the overall implementation of COVID-19 protection protocols in general and the effect of each item in particular. Hence, the model summary Table of multiple regression analysis as indicated in Appendix 1 shows that only 35.5% of the variation in the overall implementation was affected by school community-related factors. Additionally, the ANOVA Table of multiple regression (p < 0.05) shows that the combined effect of the 9 school community-related factors (Table 6 above) on the overall implementation of COVID-19 protection protocol was statistically significant. More specifically, the coefficients Table indicates that item numbers 2 (school community reluctance), 3 (inability to keep physical distancing), and 8 (absence of awareness-raising mechanisms) are statistically affecting the implementation of the COVID-19 protection protocol.

## 5 Conclusions

The findings of the study indicated that the overall mean score of the 15 items prepared with the objective of determining the implementation of the COVID-19 protection protocols was 2.38, which is near the average mean score. This indicates that the implementation of the COVID-19 protection protocols in schools is below the standard set by MoE. Hence, the COVID-19 task force needs to be reinitiated and follow-up strategies need to be strengthened to enhance the implementation status.

In addition, as confirmed in the finding part of the study, both school community-related and resource-related factors are highly affecting the implementation of the COVID-19 protection protocols with an overall mean score of 2.97 and 2.67 respectively.

However, multiple regression tests were conducted to identify the factors which were significantly affecting the implementation of COVID-19 protection protocol and the result indicated that school community reluctance (Beta = 0.29, p < 0.05) and inability to keep physical distancing principle (Beta = 0.34, p < 0.05) among the school community-related factors and deficiency of body temperature measuring device (Beta = 0.25, p < 0.05) among resource-related factors are significantly affecting the implementation of COVID-19 protection protocols. Hence, resources like water, sanitizers, thermometers, desks, and others that are important for keeping human hygiene in one way and preventing COVID-19 in other way need to be sufficiently availed in schools through getting a discussion with various local, national, and international agencies that work on the prevention of COVID-19.

## 6 Recommendations

Based on the above conclusions, which were drawn from the results and discussion of the study, the following suggestions were recommended for future improvement:

(1) MoH and MoE jointly provide orientation and short-term training for stakeholders like school community, COVID-19 task forces and school administrative bodies to scale-up their skills and understanding as well as to create conducive teaching and learning environment.
(2) MoH, MoE and Werabe administrative town advised to fulfill limited resources like water, sanitizers, thermometers, desks to minimize possibility of spread-out of COVID-19 in the schools
(3) School community reluctance needs to be avoided by creating discussion forums with the school community and enforcing rules and regulations about the COVID-19.
(4) Encouraging communication and coordination among education actors, families, learners, and the health community needs to get a priority. To explain this more, the problems experienced by both teachers and school administrators on teaching and learning during COVID-19 pandemic may be resolved through colleague assistance and team work (Karakose et al., 2022).

## 7 Limitations of the study and implication for future research

One of the main limitations of the current research has been that the data was collected only from Werabe administrative town of Silte zone. Additionally, the health professionals of Werabe administrative town were not taken into account for the COVID-19 protection protocols. The generalizability of the results is also limited because the research sample consisted only teachers, principals and supervisors. The other limitation of the study is that it did not incorporate community at large and beneficiaries of the COVID-19 protection protocols. Thus, these all might affect the findings of this study. Consequently, it is recommended that more detailed studies can be conducted in the future in different zones as well as in the entire country.

Hence, the present study has tried to address variables like the implementation of COVID-19 protection protocols and the challenges affecting its implementation. However, it doesn't address factors like the effect of the COVID-19 on students' academic achievement, teaching and assessment methods applied by teachers in the classroom, the psychological makeup of students, etc. Hence, future researchers are required to focus on these areas.

## Declarations

### Author contribution statement

Birhanu Sintayehu: Performed the experiments; Analyzed and interpreted the data; Contributed reagents, materials, analysis tools or data; Wrote the paper.

Anwar Ahmed: Conceived and designed the experiments; Performed the experiments; Analyzed and interpreted the data; Contributed reagents, materials, analysis tools or data; Wrote the paper.

### Funding statement

This research did not receive any specific grant from funding agencies in the public, commercial, or not-for-profit sectors.

### Data availability statement

Data included in article/supplementary material/referenced in article.

### Declaration of interests statement

The authors declare no conflict of interest.

### Additional information

No additional information is available for this paper.

## Appendix A Supplementary data

Supplementary File _spl_Appendix part_spl_ Supplementary File _spl_Appendix part_spl_
