# Supplementary material for: Implementation of Covid-19 protection protocols and its implication on learning & teaching in public schools
Source: Heliyon. 2022 May 2;8(5):e09362. doi: 10.1016/j.heliyon.2022.e09362 (PMC9057939; doi:10.1016/j.heliyon.2022.e09362)
Supplement: Supplementary File _spl_Appendix part_spl_ [file mmc1.docx]

**Appendix-1**

**Werabe University**

**School of Education and Behavioral Sciences**

**Questionnaire to be filled by Teachers**

**Dear Teachers:**

The main purpose of this questionnaire is to gather information for research entitled “**Implementation of COVID-19 Protection Protocols and Its Implication on Learning & Teaching Process of Public Schools”.** So you are kindly requested to answer the questions honestly. Also note that your responses will be used for only academic purpose. All the responses will be kept strictly confidential and completely anonymous. If you have any queries, please contact the researcher using mob numbers 0939418807 or 0912197349.

Thank you in advance for your time and willingness to share your ideas in this study.

1. **Background Information of the respondents**
2. School Name _______________________
3. Sex ______
4. Age ________
5. Your teaching experience in years _____________
6. Area of your specialization/training (Circle one below)
7. Natural Science
8. Social Science
9. Your current level of Education (Circle one below)
10. Diploma

b. BA/BSc

c. MA/MSc/MED

d.Others, Please specify _________________

**Part I: Items related with the extent to which schools implement COVID-19 protection protocols.**

Please rate your responses by putting “✓” mark in the appropriate column.

**Key to numbers**

0 –Never; 1 – Rarely 2 – Sometimes; 3 – Usually; 4 – Always

| **No** | **School Level Activities:** My school | **Scale** | | | | |
| --- | --- | --- | --- | --- | --- | --- |
|  |  | **0** | **1** | **2** | **3** | **4** |
| 1 | Provide awareness about the impact of COVID-19 for the students and staff through discussion forums |  |  |  |  |  |
| 2 | Applied the appropriate physical distancing principles (minimum 1 meter) |  |  |  |  |  |
| 3 | Ensured appropriate use of masks where physical distancing cannot be maintained |  |  |  |  |  |
| 4 | Clean and disinfect frequently touched surfaces such as door handles, desks, toys, supplies, light switches, doorframes, play equipment, teaching aids used by children and covers of shared books. |  |  |  |  |  |
| 5 | Allocated only 20-25 student per classroom |  |  |  |  |  |
| 6 | Applied the principle of “one student per desk” |  |  |  |  |  |
| 7 | Availed sufficient water in order to help students and staff to keep themselves clean (hygiene) |  |  |  |  |  |
| 8 | Prepared thermometer to measure body temperature of staff at the gate or entrances |  |  |  |  |  |
| 9 | Practiced one book per student principle |  |  |  |  |  |
| 10 | Differentiated the gates (entrances) to allow students to get in and out (If shifts are available) or differentiated exit and entrance gate |  |  |  |  |  |
| 11 | Made classrooms to have windows that ventilate sufficient air in order to avoid suffocation |  |  |  |  |  |
| 12 | Prepared alternative (extra) classrooms to overcome the challenges if symptoms of COVID-19 is happened |  |  |  |  |  |
| 13 | Established clear information and feedback sharing mechanisms with parents, students and teachers |  |  |  |  |  |
| 14 | provide sufficient soap and clean water or alcohol-based rub at school entrances and throughout the school |  |  |  |  |  |
| 15 | Designed continuous follow up or support mechanisms in relation to COVID 19 |  |  |  |  |  |

**Part 2: Resource related factors affecting the implement COVID-19 protection protocols.**

Please rate your responses by putting “✓” mark in the appropriate column.

**Key to numbers**

0 –Not at all; 1–to some extent 2–moderately; 3 – Highly; 4 – Extremely

| **No** | **School Level Activities:** My school | **Scale** | | | | |
| --- | --- | --- | --- | --- | --- | --- |
|  |  | **0** | **1** | **2** | **3** | **4** |
| 1 | Scarcity of chemicals to clean the school |  |  |  |  |  |
| 2 | Shortage of mask to cover the mouth and nose |  |  |  |  |  |
| 3 | Shortage of materials like soap, alcohol sanitizer |  |  |  |  |  |
| 4 | Shortage of water |  |  |  |  |  |
| 5 | Deficiency of body temperature measuring device (Thermometer at gates) |  |  |  |  |  |
| 6 | Shortage of textbooks to apply the principle of one book for one student |  |  |  |  |  |
| 7 | Shortage of classrooms |  |  |  |  |  |
| 8 | Shortage of chairs and desks |  |  |  |  |  |
| 9 | Shortage of books to practice one book one student principle |  |  |  |  |  |
| 10 | Uncomfortable classroom structure to ventilate air |  |  |  |  |  |
| 11 | Shortage of teachers |  |  |  |  |  |

**Part 3: School community related factors affecting the implement COVID-19 protection protocols.**

Please rate your responses by putting “✓” mark in the appropriate column.

**Key to numbers**

0 –Not at all; 1–to some extent 2–moderately; 3 – Highly; 4 – Extremely

| **No** | **School Level Activities:** My school | **Scale** | | | | |
| --- | --- | --- | --- | --- | --- | --- |
|  |  | **0** | **1** | **2** | **3** | **4** |
| 1 | Lack of awareness about COVID 19 |  |  |  |  |  |
| 2 | School community reluctance towards COVID 19 protocols |  |  |  |  |  |
| 3 | Inability to keep physical distancing |  |  |  |  |  |
| 4 | Carelessness in using masks |  |  |  |  |  |
| 5 | Teaching methods used by teachers in classrooms |  |  |  |  |  |
| 6 | Assessment techniques practiced by teachers |  |  |  |  |  |
| 7 | Types of play practiced by students |  |  |  |  |  |
| 8 | Absence of awareness-raising mechanisms by the school management |  |  |  |  |  |
| 9 | Absence of progress follow-up concerning the practice of protection protocols |  |  |  |  |  |

**Appendix-II**

**Questions for interview guide (For zonal/woreda heads)**

1. When was teaching learning started in school of werabe administrative town? did the schools fulfilled the standard set before re-opening?
2. Is there COVID-19 task force at administrative town, werada, kebele and school level? who chairs, who are the members? did they started their work? What do they do?
3. What is the role of task force at school, werade and town level?
4. What does the follow up strategy look like at present moment?
5. What is the role of water, finance, health bureau, women and youth affairs office for schools?
6. What are the challenges in relation to COVID-19 protection protocol implementation?

**Appendix III- Observation checklist**

| **No** | **Description** | **Yes/No** | **Recommendation** |
| --- | --- | --- | --- |
|  | **Inside the classroom** |  |  |
| 1 | One student per desk principle applied in school |  |  |
| 2 | The classroom is made to have only 20-25 student per classroom |  |  |
| 3 | One book per student applied in school |  |  |
| 4 | Students and teachers wear masks appropriately |  |  |
| 5 | Appropriate physical distancing principles (minimum 1 meter) is applied in classroom |  |  |
| 6 | The classroom is sufficiently ventilated |  |  |
| 7 | Teachers use appropriate teaching methods that reduce students contact |  |  |
| 8 | Different awareness raising posters are posted in classroom |  |  |
|  | **Outside the classroom** |  |  |
| 1 | Entry and exit gates are separated |  |  |
| 2 | water, sanitizers, mask are sufficiently available |  |  |
| 3 | Appropriate physical distancing principles (minimum 1 meter) while playing in schools |  |  |
| 4 | Prepared thermometer to measure body temperature of staff at the gate or entrances |  |  |
| 5 | Different awareness raising posters are posted in school compound |  |  |
